# Temperature and Physicochemical Properties of Abnormal Heating Composite Insulators

**DOI:** 10.3390/polym16213010

**Published:** 2024-10-26

**Authors:** Li Wang, Qin Hu, Xing Xu, Lipeng Rao, Xingliang Jiang

**Affiliations:** Xuefeng Mountain Energy Equipment Safety National Observation and Research Station, Chongqing University, Chongqing 400044, China; 20211101041@cqu.edu.cn (L.W.); 202211021025@stu.cqu.edu.cn (X.X.);

**Keywords:** abnormal heating, composite insulators, physicochemical properties, ambient humidity, insulator aging

## Abstract

Abnormal heating will reduce the insulation performance of composite insulators or even cause insulator fracture, and the abnormal heating phenomenon is more serious under high-humidity conditions. Therefore, in this paper, the voltage withstand test of abnormal heating composite insulators caused by aging and damp sheath, decay-like core rod, and contamination were carried out under different ambient humidity. The heating and discharge of composite insulators were observed by infrared thermal imager and ultraviolet imager, and its temperature characteristics were analyzed from the aspects of heating range, heating shape, and temperature difference. In addition, in order to study the abnormal heating mechanism of composite insulators, the micro-morphology, chemical groups and dielectric properties of the silicon rubber of composite insulators with aging and damp sheath and the decay-like core rod were also measured. It is found that the temperature characteristics of the three types of abnormal heating composite insulators are different, and the temperature difference increases with the increase of humidity. The deterioration of silicone rubber and core rod material is the internal reason for the abnormal heating of composite insulators, and high-humidity conditions will exacerbate the heating phenomenon.

## 1. Introduction

Abnormal heating has been the most frequent failure of composite insulators in the power grid in recent years [1]. In December 2016, Henan Power Grid conducted infrared inspections on three 500 kV lines and found that 52 composite insulators had heating defects. In February 2018, Guangdong Power Grid found that 48 composite insulators had abnormal heating during the inspection of the Haiguo Line, accounting for 11.4% of the total composite insulators of the line. In 2019, the 500 kV Tianou/Tianmu line of Zhejiang Power Grid was disconnected, and a large number of composite insulators in the line were heated [2]. In 2020, a 220 kV line in Guizhou Power Grid experienced internal breakdown of composite insulators, and composite insulators in the same batch experienced large-scale heating. Abnormal heating will cause the insulation performance of composite insulators to decline or even cause insulator fracture, which will seriously disturb the stability of the power system [3]. Therefore, it is of great significance to study abnormal heating composite insulators.

Infrared temperature measurement technology is currently one of the most widely used online non-destructive testing technologies in operation and maintenance departments. According to the standard DL/T 664-2016, the temperature difference of the composite insulator needs to be below 1 K, which means that the composite insulator is required to hardly generate heat in actual operation [4]. However, in the high-humidity area in the south, the normal operation of the composite insulator will also have a temperature rise phenomenon, which leads to the false detection of the infrared temperature detection technology.

For the heating of composite insulators caused by aging and damp sheath, Gan Yongye [5] concluded that the heat-generating region of composite insulators with aging and dampness of the sheath is usually located between the high-voltage end fittings and the second large umbrella skirt. Wang Liming et al. [6] found that aged silicone rubber becomes more prone to moisture absorption, with increased polarization loss causing heating of the composite insulator’s high-voltage end sheath. Tu et al. [7,8,9] suggested that silicone rubber aging is the internal cause of abnormal heating, while high-humidity serves as the external cause. For the heating of composite insulators caused by decay-like core rod, Wang et al. [10] proposed that boundary defects between the core rod and sheath can cause partial discharge, leading to abnormal temperature rise in the composite insulator. Zeng et al. [11] found that the reasons for the abnormal heating of decay-like composite insulators are complex, including polarization loss, partial discharge, and resistance loss caused by the deterioration of the core rod. For the heating of composite insulators caused by surface contamination, Edson G et al. [12] found that surface contamination causes abnormal heating at the high-voltage end of composite insulators. Wang et al. [13] found that with the increase of the degree of pollution, the heating phenomenon becomes more and more serious, and the ambient humidity is very important to its abnormal temperature rise.

At present, scholars at home and abroad have measured the heating amplitude of abnormal heating composite insulators [14,15], but have not considered its heating range and heating shape. A certain branch of abnormal heating composite insulators has been studied, but the temperature characteristics of different types of abnormal heating composite insulators have not been compared and analyzed. In addition, few scholars have analyzed the physical and chemical properties of the aged silicone rubber and the decay-like core rod material of the composite insulator with abnormal heating, which helps to speculate the heating mechanism of the composite insulator.

In this paper, the withstand voltage test of three types of abnormal heating composite insulators under different ambient humidity is carried out in a large multi-functional artificial climate chamber. The heating and discharge of composite insulators were observed with infrared thermal imager and ultraviolet imager, and its temperature characteristics were analyzed from the aspects of heating range, heating shape, and temperature difference. At the same time, changes in the microstructure of abnormally heated composite insulators are studied by electron scanning microscopy; changes in the chemical groups of composite insulators are studied by Fourier infrared spectroscopy; and changes in the dielectric constant of composite-insulation insulators are studied by wide-frequency dielectric spectrometry, which analyzes the thermal effects caused by partial discharges.

## 2. Materials and Methods

### 2.1. Test Samples and Test Device

In this paper, 12,220 kV composite insulators from China Pinggao Group Co., Ltd. ( Pingdingshan City, Henan, China) were used and showed abnormal heat generation., which were six composite insulators with aging and damp sheath and six decay-like composite insulators. The solid coating method [16] was used to artificially construct two surface-area fouling composite insulators, and the salt deposition density per unit area on the insulator surface (referred to as salt density) was 0.08 mg/cm^2^ and 0.12 mg/cm^2^, respectively, and the ratio of the salt density to the dust deposition density per unit area of the insulator surface (referred to as gray density) was 1:6. Sodium chloride is used to simulate conductive substances and diatomaceous earth to simulate insoluble substances. The operated insulators are susceptible to contamination, and low amplitude heating occurs at the high-voltage end of the composite insulators when the sheath is aged. Therefore, two aging and damp composite insulators were cleaned and then manually coated with dirt.

**Figure 1:**
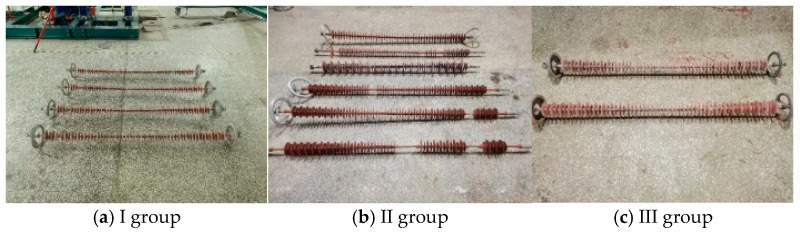
Abnormal heating composite insulator samples.

The three types of abnormal heating composite insulator samples were divided into three groups, numbered I~III groups. Group I represents the composite insulator with aging and damp sheath, group II represents the decay-like composite insulator, and group III represents the contaminated composite insulator. Insulators in each group of patterns are numbered sequentially. Figure 1 shows the general situation of the composite insulators with aging and damp sheath, decay-like composite insulators and contaminated composite insulators.

**Figure 2:**
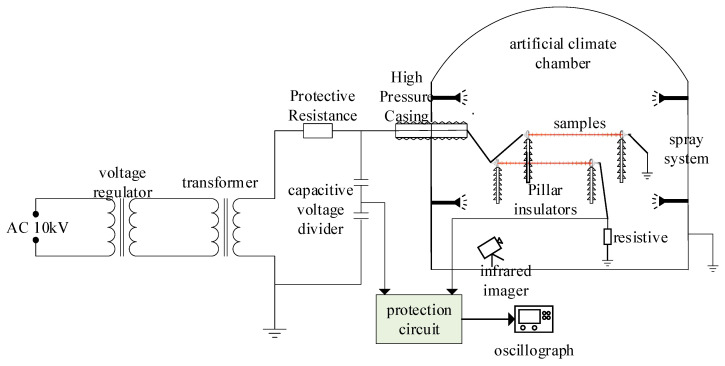
Schematic of temperature characteristic tests of abnormal heating composite insulators.

The abnormal heating composite insulator temperature characteristic test was carried out in a large-scale multi-functional artificial climate laboratory, and the test principle is shown in Figure 2. In order to avoid the umbrella skirt blocking and infrared camera (FLIR T650sc) shooting angle limitations, the test will be anomalous heat composite insulators placed on the top of the pillar insulators for access, to ensure that the sheath surface-temperature measurement precision. At this time, the infrared thermal imager can completely photograph the entire composite insulator, and can avoid the measurement deviation caused by the shielding problem of the shed when the vertical suspension is connected. Since the research contents of similar abnormal heating insulators were the same, two abnormal heating composite insulators were selected in parallel each time, and the temperature characteristic test was carried out at the same time.

### 2.2. Test Methods

In order to study the temperature characteristics of three non-normally heated composite insulators under different humidity environments, the RH (Relative humidity) in the room was adjusted to 50%, 70%, 80%, and 90%, respectively. Subsequently, the heat generation of these insulators was observed under voltage withstand test conditions. The temperatures in the climatic test room and environment were close and were within 10–15 °C.

(1) Place the abnormally heated composite insulator in the artificial climate chamber, support it with pillar insulators, and connect the circuit according to Figure 2. Adjust the ambient humidity in the artificial climate chamber, and maintain the ambient humidity unchanged for 1 h, so that the anomalous heat composite insulator is fully moist.
(2) Utilize the AC test transformer to pressurize the abnormally heated composite insulator. Taking into account the operating margin, the added voltage is 1.1 times the actual operating voltage of the composite insulator, and the abnormal heating composite insulator is found after 2 h of voltage withstand temperature measurement.
(3) After the temperature rise of the composite insulator is stable, the abnormal heating composite insulator is photographed with an infrared thermal imager, and the maximum and minimum surface temperatures of composite insulator are recorded, respectively. According to the standard DL/T 664-2016 the temperature difference of the composite insulators (defined as the difference between the surface temperatures of different parts of the same device under test) must not exceed 1 K [17], so the temperature difference of composite insulators with abnormal heating is defined as $\Delta T=T_{\max}-T_{\min}$, and a K. A UV imager is used to record a 1 min video to observe the discharge of an abnormally hot composite insulator.

## 3. Influence of Humidity on Temperature Characteristics of Abnormal Heating Composite Insulator

### 3.1. Composite Insulator with Aging and Damp Sheath

**Figure 3:**
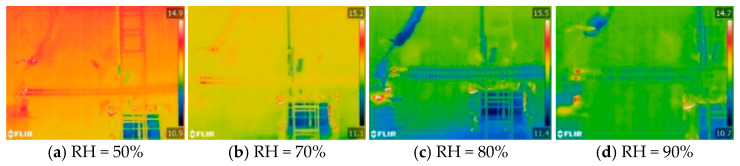
Infrared thermal images of I-1 and I-2 under different humidity.

The infrared thermal images of I-1 and I-2, after stable temperature rise under different ambient humidity, are shown in Figure 3. In the infrared thermal image, the upper insulator is I-1, and the lower insulator is I-2. It can be seen from the figure that the heating range of the composite insulators with aging and damp sheath is at the high-voltage end, while there is no abnormal heating phenomenon in the middle- and low-voltage ends. Due to the short heating interval, the heating shape of the composite insulators with aging and damp sheath can be regarded as dot heating relative to the length of the entire insulator. When the relative humidity is 50%, the heating range of the composite insulators is from the high-voltage end fittings to the first large-shed group. When the relative humidity increases to 90%, the abnormal heating phenomenon occurs between the first large-shed group and the second large-shed group of the composite insulators, indicating that the increase of humidity will cause an increase of the heating range of composite insulators with aging and damp sheath.

**Figure 4:**
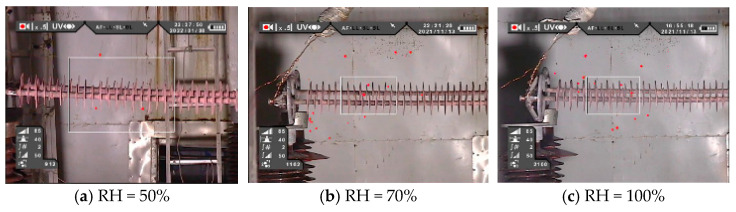
UV images of I-1 and I-2 under different humidity.

The UV imaging images of I-1 and I-2 at relative humidity of 50%, 70%, and 90% are shown in Figure 4. It can be seen from the figure that there is no obvious discharge phenomenon at the high-voltage end of the composite insulators, but there is some stray corona and, with the increase of humidity, the stray corona increases slightly. It can be concluded that the abnormal heating of the composite insulators with aging and damp sheath is not related to partial discharge.

Table 1 shows the calculation results of the temperature difference of the composite insulators with aging and damp sheath under different environmental humidity. The temperature differences of the composite insulators with aging and damp sheath under a relative humidity of 50% are 0.8 K, 0.6 K, 0.9 K, and 0.7 K, respectively, and the temperature differences are all less than 1 K, indicating that under low-humidity conditions, the four composite insulators meet the standard DL/T 664-2016 requirements. However, with the increase of humidity, the heating of the composite insulators with aging and damp sheath becomes more and more serious. When the relative humidity increased to 90%, the temperature difference of I-1, I-3, and I-4 were greater than 2 K, and the temperature difference of I-3 was as high as 4.3 K. As the ambient humidity increases, the run-aged silicone rubber surface loses its hydrophobicity and is more susceptible to wetting behaviors, resulting in an increase in the polarization loss of the high-voltage end under the action of the electric field, so the heating of the composite insulator intensifies.

The dielectric loss heating power *p*_1_ caused by the polarization effect of the composite insulator under the action of the alternating electric field can be expressed by the following formula:

(1) $$p_{1}={U^{2}}_{d}\omega C\tan\delta$$

where *U_d_* refers to the distributed voltage of the composite insulator, and the unit *V* is the voltage angular frequency, and the unit is rad/s; *C* is the equivalent capacitance of the composite insulator, and the unit is F, tanδ is the dielectric loss factor at operating temperature. It can be seen from the measurement results of the dielectric parameters in the fourth section of the paper that the dielectric loss factor of the aged silicone rubber increases sharply after being dampened, resulting in an increase in the heating power and temperature.

### 3.2. Decay-like Composite Insulators

**Figure 5:**
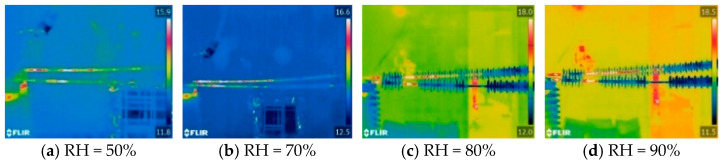
Infrared thermal images of I-1 and I-2 under different humidity.

The infrared thermal images of II-1 and II-2 after a stable temperature rise under different ambient humidity are shown in Figure 5. In the infrared thermal image, the upper insulator is II-1, and the lower insulator is II-2. It can be seen from the infrared thermal image that the heating range of the decay-like composite insulators is wide, and the high-voltage, middle- and low-voltage end of the insulator may have abnormal heating phenomenon. Taking the heating range of the decay-like composite insulator under RH = 70% as an example, the heating range of II-1 and II-2 accounts for about 1/2 of the length of the whole insulator, and the low-voltage end of II-2 also has abnormal heating. It can be found that the heating range of the decay-like composite insulator is much larger than that of the composite insulators with aging and damp sheath. Due to the long heating range of the composite insulators, the heating shape of the decay-like composite insulator can be regarded as segment heating relative to the length of the entire composite insulator.

Table 2 shows the calculation results of the temperature difference of the decay-like composite insulator under different humidity. It can be seen from the table that when the relative humidity is 50%, the heating of the mandrel-rotten composite insulator is extremely serious, and the average temperature difference reaches 17.9 K. In the test, the temperature difference of five composite insulators was higher than 10 K, and the temperature difference of II-3 and II-6 reached 36.7 K and 21.7 K respectively. Under low-humidity conditions, the temperature differences of the composite insulators with aging and damp sheath were all less than 1 K, while the decay-like composite insulator has obvious heating. Therefore, the temperature difference under low-humidity conditions is an important feature that distinguishes the decay-like composite insulator from the composite insulators with aging and damp sheath. The heating caused by polarization loss and leakage current under low-humidity conditions can be ignored, indicating that the defect of the core rod itself is the root cause of its abnormal heating, rather than the false heating caused by the increase of ambient humidity.

**Figure 6:**
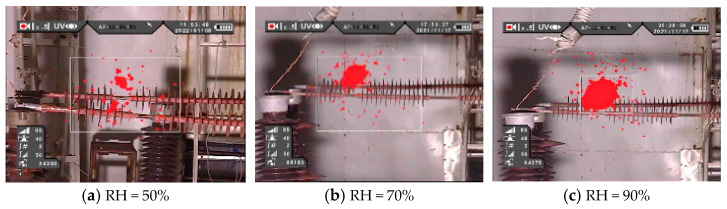
UV images of II-1 and II-2 under different humidity.

The UV imaging images of II-1 and II-2 at relative humidity of 50%, 70%, and 90% are shown in Figure 6. It can be seen from the UV imaging image that the decay-like composite insulator has obvious partial discharge phenomenon. Combined with the infrared thermal image, it is found that where the partial discharge is most obvious, the heating phenomenon of the decay-like composite insulator is the most serious, which indicates that the partial discharge is the main reason for the heating of the decay-like composite insulator.

**Figure 7:**
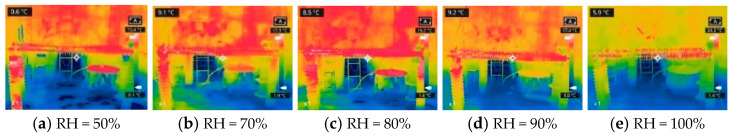
Infrared thermal images of III-1 and III-2 under different humidity.

The infrared thermal images of III-1 and III-2 after stable temperature rise under different ambient humidity are shown in Figure 7. In the infrared thermal image, the upper insulator is III-1, and the lower insulator is III-2. It can be seen from the figure that when the relative humidity is 80% and below, the heating range of the contaminated composite insulator is concentrated on the high-voltage side of the insulator, and there is no abnormal heating phenomenon on the low-voltage side. When the relative humidity increased to 90% and 100%, different degrees of heating occurred everywhere in the composite insulator. When the relative humidity increased to 90% and above, the contamination on the surface of the composite insulator was fully wetted, and the surface current increased sharply, resulting in abnormal heating of the composite insulator. When the relative humidity was 70%, the heating range of III-1 is from the high-voltage end fitting to the 15# shed, and the heating range of III-2 was the high-voltage end fitting to the 18# shed. It can be seen that under the same ambient humidity, the more contaminated the surface of the composite insulator, the longer the heating range. From the point of view of the heating shape, the contaminated composite insulator can also be regarded as segment heating.

Table 3 shows the calculation results of the temperature difference of the contaminated composite insulator under different humidity. It can be seen from the table that under the same ambient humidity, the temperature difference of III-2 is higher than that of III-1, indicating that the temperature difference will increase with the increase of equivalent salt deposit density. When the relative humidity is 50%, the average temperature difference of the contaminated composite insulator is about 5 K, which is higher than that of the composite insulators with aging and damp sheath. With the increase of ambient humidity, the temperature difference of the contaminated composite insulator gradually increased. When the relative humidity was increased to 70%, 80%, 90%, and 100%, the average temperature differences of the contaminated composite insulators increased to 7.5 K, 8.4 K, 10.5 K, and 14.4 K, respectively. Under dry conditions, the surface pollution resistance of the composite insulator is large and the leakage current is small, so the temperature difference is small. However, the increase of humidity makes the moisture in the air constantly invade the surface pollution, which gradually increases the leakage current and the temperature difference.

The heating power *P*_2_ caused by the surface leakage current of composite insulator under the action of alternating electric field can be expressed by the following formula:

(2) $$P_{2}=U_{d}I_{g}$$

where *U_d_* refers to the distributed voltage of composite insulator, and the unit is V; *I_g_* is the resistive component of leakage current of composite insulator, and the unit is A.

As the voltage amplitude increases, the resistive component *I_g_* of the leakage current on the surface of the composite insulator increases, which also causes the distribution voltage at each place of the insulator to increase, so the heating power is greatly increased, which makes the temperature difference of the composite insulator increase gradually.

**Figure 8:**
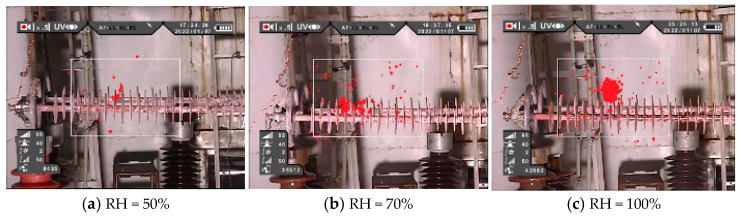
UV images of III-1 and III-2 under different humidity.

The UV imaging images of III-1 and III-2 at relative humidity of 50%, 70%, and 100% are shown in Figure 8. When the relative humidity is 50%, a weak partial discharge phenomenon occurs between the 7~9# sheds of the contaminated composite insulators. When the relative humidity increased to 70%, the partial discharge phenomenon was significantly enhanced. Combined with the infrared thermal images in Figure 7a,b, it can be seen that the sheath between the 7~9# sheds has abnormal heating, but it is not the most serious position of heating, indicating that partial discharge is one of the reasons for abnormal heating of contaminated composite insulators, but it is not the most fundamental reason.

## 4. Physicochemical Properties of Abnormal Heating Composite Insulators

The aging of the silicone rubber material is the internal reason for the abnormal heating of the composite insulators with aging and damp sheath. Therefore, for the composite insulators with aging and damp sheath, this paper focuses on the physicochemical properties at the heating area of the high-voltage end sheath.

### 4.1. Study on the Physicochemical Properties of the High-Voltage and Sheath

**Figure 9:**
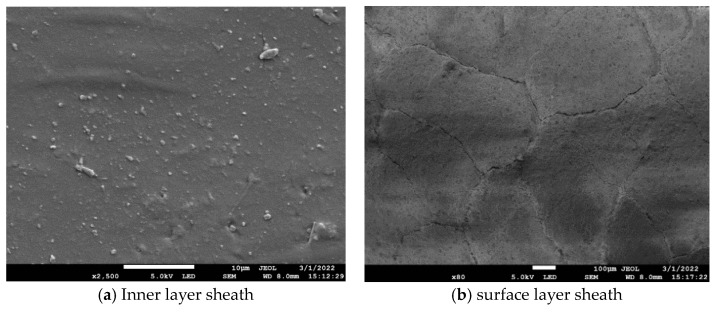
Micrographs of inner and outer sheaths at the heating area.

JEOL JSM-7800F (Nippon Electronics Corporation, Yokoguchi, Japan) field emission scanning electron microscope was used to observe the surface and inner layer sheaths at the heating area of the high-voltage end of I-1, and the results are shown in Figure 9. The figure shows that there is no crack in the inner layer of the silicone rubber sample at the heating place, and the surface structure remains smooth and the roughness is small. Because filler particles were added in the preparation of silicone rubber, micron-sized particles were observed to be uniformly distributed on the surface of the silicone rubber, from which it can be inferred that the inner layer sheath of the high-voltage end of the composite insulators with aging and damp sheath does not appear aging. The surface layer of the silicone rubber sample at the heating area is full of cracks and is divided into irregular loose block structures by the cracks. The area around the crack is uneven and there are a large number of holes formed by deterioration, indicating that the aging of the composite insulators with aging and damp sheath mainly occurs in the surface layer.

**Figure 10:**
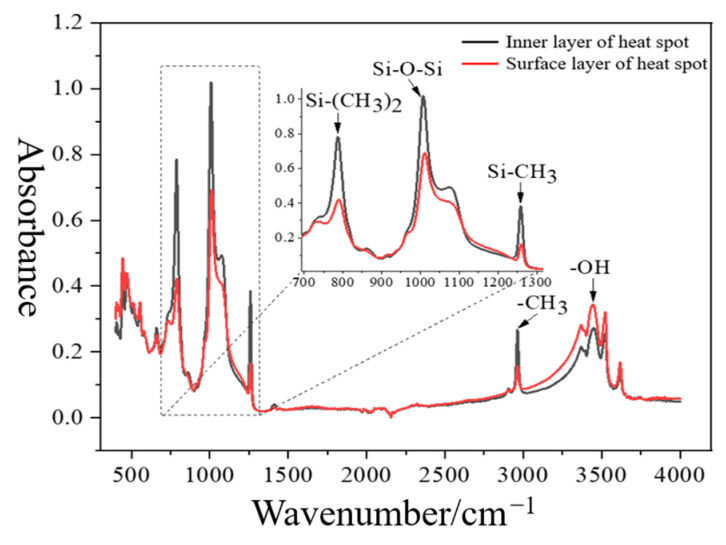
Infrared spectrum of the surface and inner sheath at the heating area.

According to GB/T 6040-2019 [18], a Nicolet iS50 Fourier transform infrared spectrometer was used to measure the surface and inner layer sheaths at the heating area of the high-voltage end of I-1, and the results are shown in Figure 10. The figure shows that, compared with the inner layer sheath, there is no new characteristic absorption peak of the surface layer sheath at the heating area, indicating that the composite insulator sheath does not generate new substances during the aging process. The contents of Si-O-Si, Si-(CH3)_2_, Si-CH3, and CH3 groups in the surface layer sheath samples at the heating area are significantly reduced. It can be inferred that the fracture degree of the main chain and side chain can characterize the aging state of insulator silicone rubber. Since the bond energy of the Si-C bond is the smallest [19], the Si-C bond is first broken during the aging process, resulting in the reduction of CH3 groups and the weakening of the hydrophobicity of the silicone rubber. The Si-O main chains without the shielding of the hydrophobic group have broken under harsh operating conditions, resulting in the degradation of PDMS molecules.

**Figure 11:**
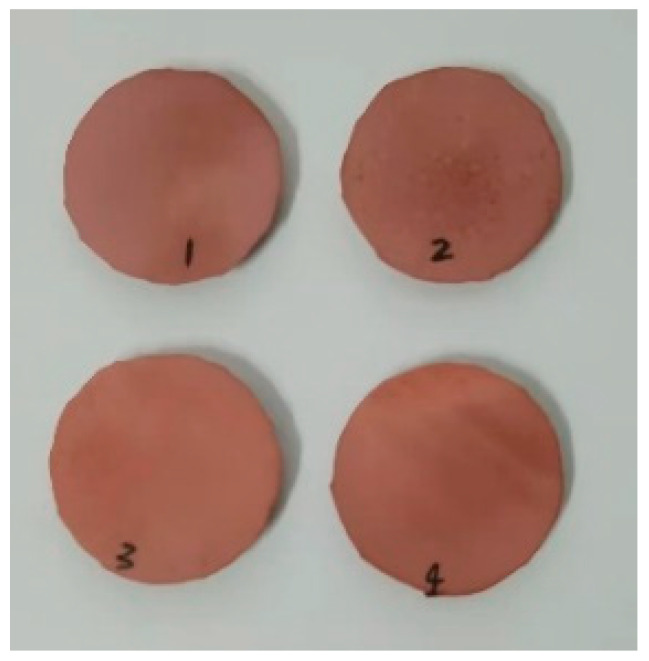
Sample for measuring dielectric properties of silicone rubber.

In this paper, Sample 1-4 with a diameter of 20 mm and a thickness of 3 mm are taken from the high-voltage, middle-, and low-voltage end of I-1~I-3, as shown in Figure 11. Novocontrol Concept 80 (Novocontrol Technologies GmbH & Co. Montabaur/GERMANY) broadband dielectric spectroscopy was used to measure the permittivity and dielectric loss factor of the samples. The average value of the measurement results of four samples of the same shed is taken as the dielectric parameter value. The measurement results of the sample at the frequency of 50 Hz and the temperature of 20 °C are shown in Table 4.

It can be seen from the table that the maximum and minimum permittivity of the three silicone rubbers are 4.54 and 4.07, respectively, and the maximum and minimum dielectric loss factors are 4.63% and 3.16%, respectively. Since I-1~I-3 are the same type of composite insulators produced by the same manufacturer, the difference in the measurement results of permittivity can indicate that the sheaths of the three composite insulators have different degrees of aging. The permittivity and dielectric loss factor of the high-voltage end silicone rubber of I-1~I-3 are larger than those of the middle- and low-voltage ends, indicating that under the action of the strong electric field at the high-voltage end, the high-voltage end silicone rubber of the composite insulator ages more seriously. At the same time, the heating position of I-1~I-3 is only at the high-voltage end, which shows that the aging of the silicone rubber material is the internal reason for the abnormal heating of the composite insulators with aging and damp sheath.

In order to study the influence of moisture on the dielectric properties of the high-voltage end silicone rubber of composite insulators, the dried high-voltage end silicone rubber samples of I-1~I-3 were placed in a constant temperature and humidity environment for 96 h. Keep the ambient temperature at 20 °C and the ambient relative humidity at 80%. The dielectric parameters were measured every 24 h, and the single measurement time was less than 3 min. The measurement results of the dielectric properties are shown in Table 5.

The table shows that the permittivity and dielectric loss factor of the dried silicone rubber are significantly reduced. The permittivity of the silicone rubber of I-2 was reduced to 3.97, and the dielectric loss factor was reduced to 1.54%, indicating that the dielectric properties of the silicone rubber were easily affected by ambient humidity. As the silicone rubber was gradually dampened, its dielectric parameters gradually increased. This is because the permittivity of water is much higher than that of silicone rubber, and the dipole polarization process intensified after the silicone rubber absorbed moisture in the air, resulting in an increase in the dielectric parameters. After being damped for 96 h, the permittivity of I-1~I-3 silicone rubber increased by 11.56% on average, and the dielectric loss factor increased by 73.6% on average. It shows that the dielectric loss factor is greatly affected by humidity, which leads to a sharp increase in the dielectric loss of the aged silicone rubber when the ambient humidity is high, which causes abnormal heating of the composite insulator. However, under low-humidity conditions, the dielectric parameters of the silicone rubber returned to normal, and the heating amplitude of the composite insulator was greatly reduced or even disappeared. Therefore, the high-humidity condition is the external reason for abnormal heating of composite insulators with aging and damp sheath.

### 4.2. Study on the Physicochemical Properties of the the Decay-like Core Rod

**Figure 12:**
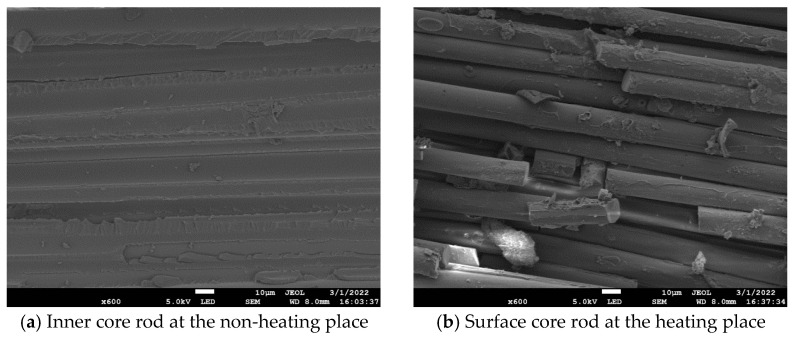
Micrographs of the core rod at the non-heating and heating places.

An electron microscope scan was carried out on the surface layer of decay-like core rod at the heating place and the inner layer at the non-heating place of II-3, and the results are shown in Figure 12. As can be seen from Figure 12a, the epoxy resin of the inner mandrel at the unheated place is smooth and flat, and the microstructure of the epoxy resin matrix and glass fiber is basically intact. The glass fiber is tightly surrounded by epoxy resin, and the interface structure between glass fiber and epoxy resin is intact. As can be seen from Figure 12b, the interface between the glass fiber and the epoxy resin of the surface layer of decay-like core rod at the heating place fails, the epoxy resin matrix basically degrades, and only a very small amount of epoxy resin remains on the surface of the glass fiber. The glass fibers are loosely arranged, and some glass fibers are broken, which will reduce the mechanical stress of the composite insulator [20].

**Figure 13:**
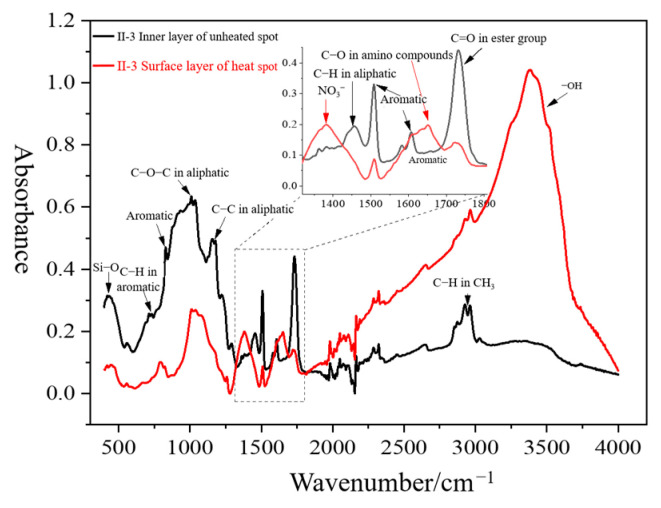
Infrared spectra of the core rod at the heating and non-heating area.

Infrared spectrum measurement was carried out on the surface layer of the decay-like core rod at the heating place and the inner layer at the non-heating place of II-3, and the results are shown in Figure 13. Compared with the infrared spectrum of the inner core rod at the non-heating place, a new absorption peak with a wavenumber of 1384 cm^−1^ appears in the infrared spectrum of the surface layer of decay-like core rod at the heating place of II-3, indicating that nitrate ions have appeared in the decay-like core rod. Since nitrogen-containing substances are not added in the manufacture of the core rod, it is speculated that the nitrate ions may be generated by partial discharge of the composite insulator in a damp environment. N_2_ and O_2_ in the air generate NO_2_ under the action of partial discharge, while NO_2_ and O_2_ dissolve in water to generate nitric acid [21]. The reaction mechanism is shown below.

(3) $$N_{2}+2O_{2}\to 2NO_{2}$$

(4) $$4NO_{2}+O_{2}+2H_{2}O\to 4HNO_{3}$$

Compared with the infrared spectrum of the inner core rod at the non-heating place, a new absorption peak with a wavenumber of 1630 cm^−1^ appeared in the infrared spectrum of the surface layer of decay-like core rod at the heating place of II-3, indicating that amino compounds appear in the decay-like core rod. It indicates that this peak was detected on infrared spectra bamboo treated with epoxy resin [22]. It can also be found that the O-H absorption peak intensity of surface layer of decay-like core rod at the heating place is significantly greater than that of the inner core rod at the non-heating place, indicating that the hydroxyl content in the decay-like core rod will increase.

Compared with the infrared spectrum of the inner core rod at the non-heating place, the absorption peak intensities at the wavenumbers of 2970~2920, 1736, 1608, 1508, 1458, 1182, and 1038 cm^−1^ decreased significantly in the infrared spectrum of the surface layer of decay-like core rod at the heating place of II-3. It shows that the content of methyl, ester, aromatic, aliphatic, and other groups in the decay-like core rod is reduced. Combined with the analysis results of electron microscopy, it can be speculated that the epoxy resin matrix in the decay-like core rod has been severely degraded under the erosion caused by partial discharge.

Compared with the infrared spectrum of the inner core rod at the non-heating place, the absorption peak intensity at the wavenumber of 480 cm^−1^ decreased in the infrared spectrum of the surface layer of decay-like core rod at the heating place of II-3, which shows that the content of the Si-O group in the decay-like core rod decreases. The Si-O group comes from the glass fiber in the core rod, so it is speculated that ion exchange and hydrolysis phenomenon have occurred in the glass fiber of the decay-like core rod [23,24].

**Figure 14:**
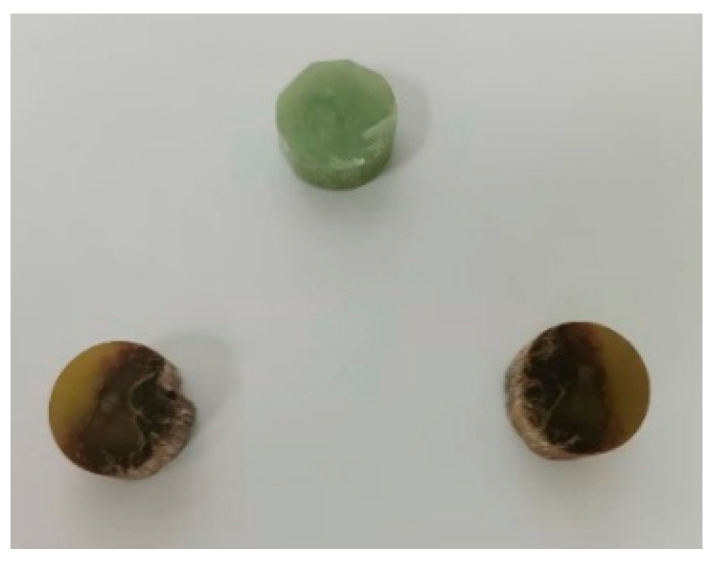
Sample for measuring dielectric properties of the core rod.

Since the measurement of dielectric properties would destroy the composite insulator specimen and the decayed mandrel is very valuable, only sample II-3 was measured, cutting the II-3 high-voltage end of the rotten mandrel and the low-voltage end of the unheated area of 18 mm diameter and 13 mm thickness of the specimen. The end of the specimen is sandpapered and smoothed, as shown in Figure 14, and the measurement results are shown in Table 6.

It can be seen from the table that the permittivity and dielectric loss factor of II-3-1 are about 4 and 26 times that of the non-heating core rod, respectively. The permittivity and dielectric loss factor of II-3-2 are about 5 and 42 times that of the non-heating core rod, respectively. The continuous deterioration of the core rod in operation leads to the generation of polar groups, resulting in the permittivity and dielectric loss factor of the decay-like core rod being much larger than that of the non-heating core rod. The large increase in the permittivity of the core rod will lead to the distortion of the electric field, which will cause serious partial discharge and heating phenomenon. If the decay-like state develops axially, internal breakdown of the composite insulator may occur. If the decay-like state develops radially, the composite insulator may break.

In order to study the influence of moisture on the dielectric properties of the decay-like composite insulator, the dried core rod samples of II-3-1 and II-3-2 were placed in an environment with a temperature of 20 °C and a relative humidity of 80% for 96 h. The dielectric parameters were measured every 24 h, and the single measurement time was less than 3 min. The measurement results of the dielectric properties are shown in Table 7.

It can be seen from the table that the permittivity and dielectric loss factor of the decay-like core rod are significantly reduced after drying. The permittivity of II-3-1 and II-3-2 dropped to 11.33 and 14.05, respectively, and the dielectric loss factors dropped to 31.91% and 45.12%, respectively, indicating that the dielectric properties of the decay-like core rod were easily affected by ambient humidity. The dielectric loss factor of the decay-like core rod after drying is still much higher than that of the non-heating core rod, indicating that the decay-like rod is seriously degraded. The permittivity and dielectric loss factor increase gradually after the mandrel is damped. After being exposed to humidity for 96 h, the permittivity of II-3-1 and II-3-2 increased by 39.67% on average, and the dielectric loss factor increased by 27.97% on average, indicating that the permittivity of the decay-like core rod is more affected by humidity. In a high-humidity environment, the dielectric constant of the corrugated mandrel increases dramatically, and the electric field distortion of the composite insulator becomes more serious, which may exacerbate the partial discharge [25], making the abnormal heating phenomenon of the corrugated composite insulator with a corrugated mandrel more serious.

## 5. Conclusions

In this paper, the temperature characteristics of various types of abnormal heating composite insulators under different ambient humidity are studied, the physicochemical properties of silicone rubber and decay-like core rod are analyzed, and the main conclusions are as follows:

(1) The heating interval of the composite insulators with surface contamination and core rod decay is much longer than that of the composite insulators with aging and dampness of the sheath, and the temperature difference between the three types of abnormally heated composite insulators increases to a different extent with the increase of humidity.
(2) The heating phenomenon is the key feature to distinguish the core rod decay, surface dirt, and sheath aging moisture composite insulators. Under low-humidity conditions, the temperature difference of sheath aging and damp composite insulators decreases or even disappears, but there is still a serious heating phenomenon in the composite insulators with corrugated mandrels and surface contamination.
(3) Sheath aging moisture composite insulator sheath aging occurs in the surface layer and the main chain, and the degree of fracture of the side chain can be used as an important parameter to characterize the aging of silicone rubber.

## Figures and Tables

**Table 1:** ΔT of composite insulators with aging and damp sheath under different humidity.

Composite Insulators	Difference in Temperature (°C)			
	RH = 50%	RH = 70%	RH = 80%	RH = 90%
I-1	0.8	1.5	1.8	2.4
I-2	0.6	1.0	1.5	1.9
I-3	0.9	1.5	2.7	4.3
I-4	0.7	1.4	1.7	2.7

**Table 2:** ΔT of decay-like composite insulators under different humidity.

Number	Difference in Temperature (°C)			
	RH = 50%	RH = 70%	RH = 80%	RH = 90%
II-1	14.9	18.7	22.7	29.3
II-2	6.1	9.3	15.9	20.5
II-3	36.7	42.9	44.2	47.2
II-4	13.8	16.2	21.5	27.4
II-5	14.2	19.2	21.6	24.9
II-6	21.7	28.3	32.3	38.3

**Table 3:** ΔT of contaminated composite Insulators under different humidity.

Number	Difference in Temperature (°C)				
	RH = 50%	RH = 70%	RH = 80%	RH = 90%	RH = 100%
III-1	4.9	6.4	7.1	8.7	11.5
III-2	5.6	8.6	9.6	12.2	17.3

**Table 4:** Measurement result of different properties of I-1~I-3 silicone rubber.

Sampling Area	I-1		I-2		I-3	
	*ε*	tan*δ*	*ε*	tan*δ*	*ε*	tan*δ*
High-voltage side	4.54	4.34%	4.43	4.54%	4.50	4.63%
Central section	4.50	4.13%	4.13	3.22%	4.22	3.47%
Low-voltage side	4.42	4.09%	4.07	3.16%	4.25	3.49%

**Table 5:** Dielectric parameters of silicone rubber after damp.

Time	I-1		I-2		I-3	
	*ε*	tan*δ*	*ε*	tan*δ*	*ε*	tan*δ*
dry	4.13	1.67%	3.97	1.54%	4.07	1.80%
24 h	4.21	2.32%	4.07	2.31%	4.28	3.68%
48 h	4.32	2.68%	4.14	3.01%	4.35	4.59%
72 h	4.43	3.59%	4.31	4.85%	4.47	6.77%
96 h	4.52	6.03%	4.48	6.02%	4.77	6.94%

**Table 6:** Measurement results of dielectric properties of I-1~I-3 silicone rubber.

Sampling Area	*ε*	tan*δ*
Unheated mandrels	8.53	2.55%
II-3-1	32.04	52.70%
II-3-2	36.09	85.52%

**Table 7:** Dielectric parameters of the core rod after damp.

Time	II-3-1		II-3-2	
	*ε*	tanδ	ε	tanδ
dry	11.33	31.91%	14.05	45.12%
24 h	13.74	33.76%	15.93	48.20%
48 h	14.53	36.14%	17.38	51.37%
72 h	17.11	40.70%	21.02	55.13%
96 h	19.27	48.29%	22.72	57.86%

## Data Availability

The original contributions presented in the study are included in the article, further inquiries can be directed to the corresponding author.
